# Evaluation of the Value of Fine Needle Aspiration Cytology and Cell Morphology in Determining the Histogenesis of Pancreatic Lesions With Review of Literature, Overview and Cytological Experience of 25 Years: Original Research

**DOI:** 10.1002/hsr2.70347

**Published:** 2025-01-12

**Authors:** Soudah Bisharah, Abbas Mahmoud

**Affiliations:** ^1^ Institute of Pathology Hannover Medical School (MHH) Hannover Germany; ^2^ Gerhard‐Domagk Institute of Pathology University Hospital Muenster (UKM) Muenster Germany

**Keywords:** cell morphology, cytology, evaluating study, FNAC, overview, pancreas lesions

## Abstract

**Background and Aims:**

Benign lesions, inflammation, cysts and pseudocysts, as well as neoplasms of the exocrine and endocrine parts of the pancreas can be easily identified using cytological methods. The sensitivity and specificity can be increased with the help of additional examination methods. The sensitivity of intraoperative rapid cytology reaches about 99%. In the literature, the sensitivity reaches an average of about 85% for biopsies. The method is easy to use, has very low complication rates (1%–2%) and is safe for the patient.

**Methods:**

1290 cytological samples from pancreatic lesions were processed in the institute of pathology at Hannover Medical School (MHH), as cytological smears and stained with Giemsa and PAS stains as conventional methods. They were compared with the histological specimens that were processed at the same institute. Immunocytochemistry and molecularpathology have been processed only in selected cases. In general, it is routine in the university that the patients give their written consent to participate in clinical studies. The local ethics committee has stated that there is no need for approval due to the retrospective nature of the study.

**Results:**

In this work, we detected 20.077% malignant lesions, 63.333% benign findings and inflammation, 7.441% pseudocysts and cysts. About 9.147% samples were unrepresentative due to insufficient number of cells.

**Conclusion:**

This work will highlight the importance of fine aspiration cytology (FNAC) of suspicious pancreatic lesions, its possibilities and limitations in routine diagnostics with discussing the differential diagnoses, pointing to its great value and safety for patients. FNAC is the gold standard, its power is strongly associated with high specificity and sensitivity in the diagnosis of pancreatic lesions, and is very useful in the differential diagnosis between malignant and inflammatory lesions in pancreas.

## 1 Introduction

The fine‐needle aspiration (FNA) of suspicious pancreatic lesions, especially of radiologically and sonographically suspicious masses, is of great importance and plays a central role in their detection. Benign lesions, inflammation, cysts and pseudocysts, as well as neoplasms of the exocrine and endocrine parts of the pancreas can be easily identified using cytological methods [1, 2]. The sensitivity and specificity can be increased with the help of additional examination methods to about 64%–94% and 71%–100%, respectively [3, 4]. The sensitivity of intraoperative rapid cytology even reaches about 99%, while in biopsies the sensitivity reaches an average of about 85% [5, 6]. The method is simple, almost without complications (1%–2%) and safe for the patient. Thirty percent of all pancreatic masses are malignant. Ductal adenocarcinoma is the most common with an incidence of > 80% and is usually only diagnosed at late stages with a poor prognosis and a 5‐year median survival of 5%. After surgical treatment, the mortality is about 20%. Chronic inflammation or autoimmune pancreatitis are important differential diagnoses. The FNA makes it possible to differentiate between the two entities and, thus, reduces the number of unnecessary operations [7, 8]. A multidisciplinary work including clinical history, imaging methods and laboratory values is needed to reach to the right diagnosis. Ancillary techniques like immunocytochemistry, and molecularpathology can be added to confirm the diagnosis [9]. A bioptic confirmation of the diagnosis (histological or cytological) is obligatory, before performing neoadjuvant therapy, regardless of whether it is a locally advanced, inoperable or metastatic pancreatic carcinoma [10, 11]. In this work, we report on our experiences with FNA of pancreatic lesions and its possibilities and limitations in routine diagnostics, taking into account the differential diagnoses.

### 1.1 Different Sections and Headlines

#### 1.1.1 Fine Needle Aspiration Cytology (FNAC)

##### 1.1.1.1 Indications of the FNA

Sonographic or radiological suspicion of a mass/pancreatic carcinoma (DD: chronic pancreatitis/cysts, necrosis, non‐Hodgkin lymphoma and metastases). Pre‐, peri‐ and postoperative.

##### 1.1.1.2 Contraindications

FNAC is contraindicated in small lesions because of sample error. It is difficult to be performed by sclerosis/fibrosis or necrosis. It is contraindicated by patient with bleeding diathesis.

##### 1.1.1.3 Contamination

The cytology from the pancreatic lesions can be contaminated from the cells of surrounding organs or from the pathway to the pancreatic lesion, such as mesothelial cells, hepatocytes, enterocytes, gastric epithelial cells or esophageal cells.

## 2 Cytological Criteria of Pancreatic Samples for Different Pancreatic Lesions Based on Our Experience

**Figure 1:**
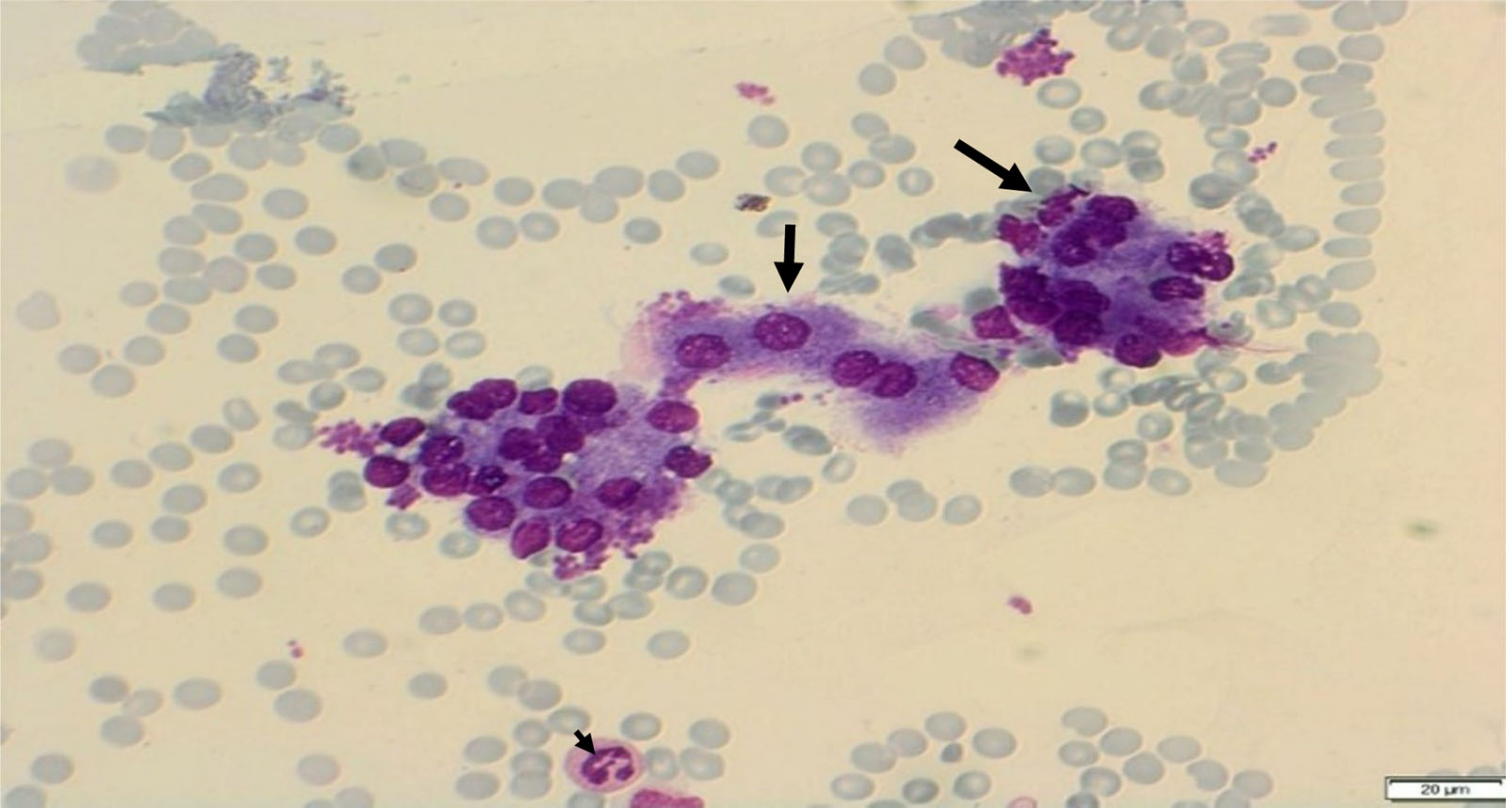
Acinar cell aggregates (long arrow), with round cell shape (short arrow), central or eccentric nucleus, homogeneous to fine granular eosinophilic cytoplasm. Neutrophilic granulocytes in the background (smaller arrow).

**Figure 2:**
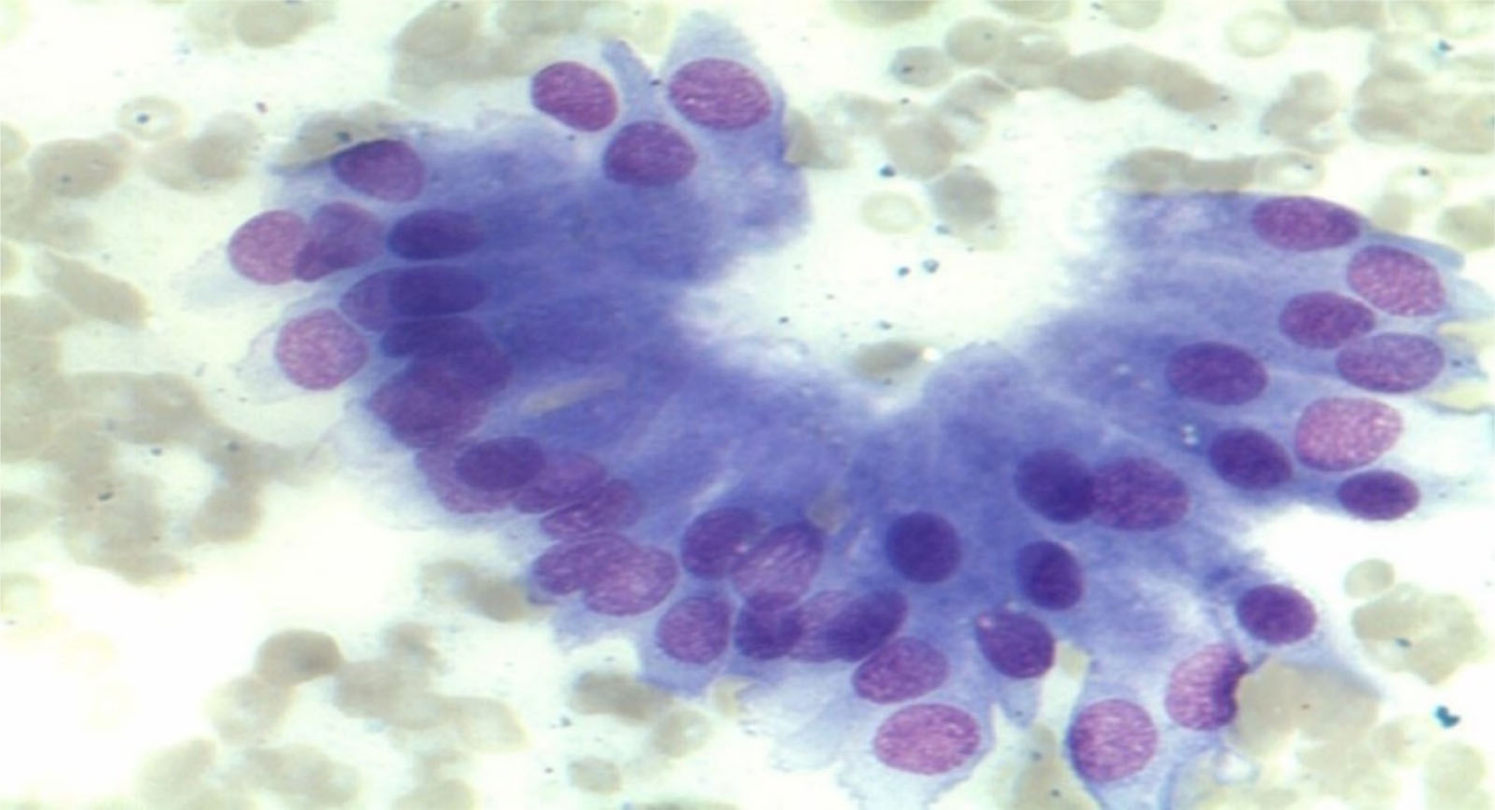
Normal cubic ductal epithelium (ductal cells).

The appearance of the normal pancreatic cells in cytology included uniform cells or acinar cell aggregates. The cells are round in shape with central or eccentric nucleus and homogenous to fine granular eosinophilic cytoplasm (Figure 1). Occasional cubic ductal epithelium (ductal cells) can be also seen (Figure 2). These cells have light blue‐stained and sparse cytoplasm. Islet cells are rarely seen, but they have bright with very faint cytoplasm and the nucleus with regularly distributed and finely granular chromatin and without nucleoli.

Benign changes of the pancreas: They are different benign lesions of the pancreas such as acute and chronic pancreatitis, malformations, lipomatous atrophy, cystic fibrosis.

## 3 Cytological Criteria of Pancreatic Samples for Different Benign Pancreatic Lesions Based on Our Experience

**Figure 3:**
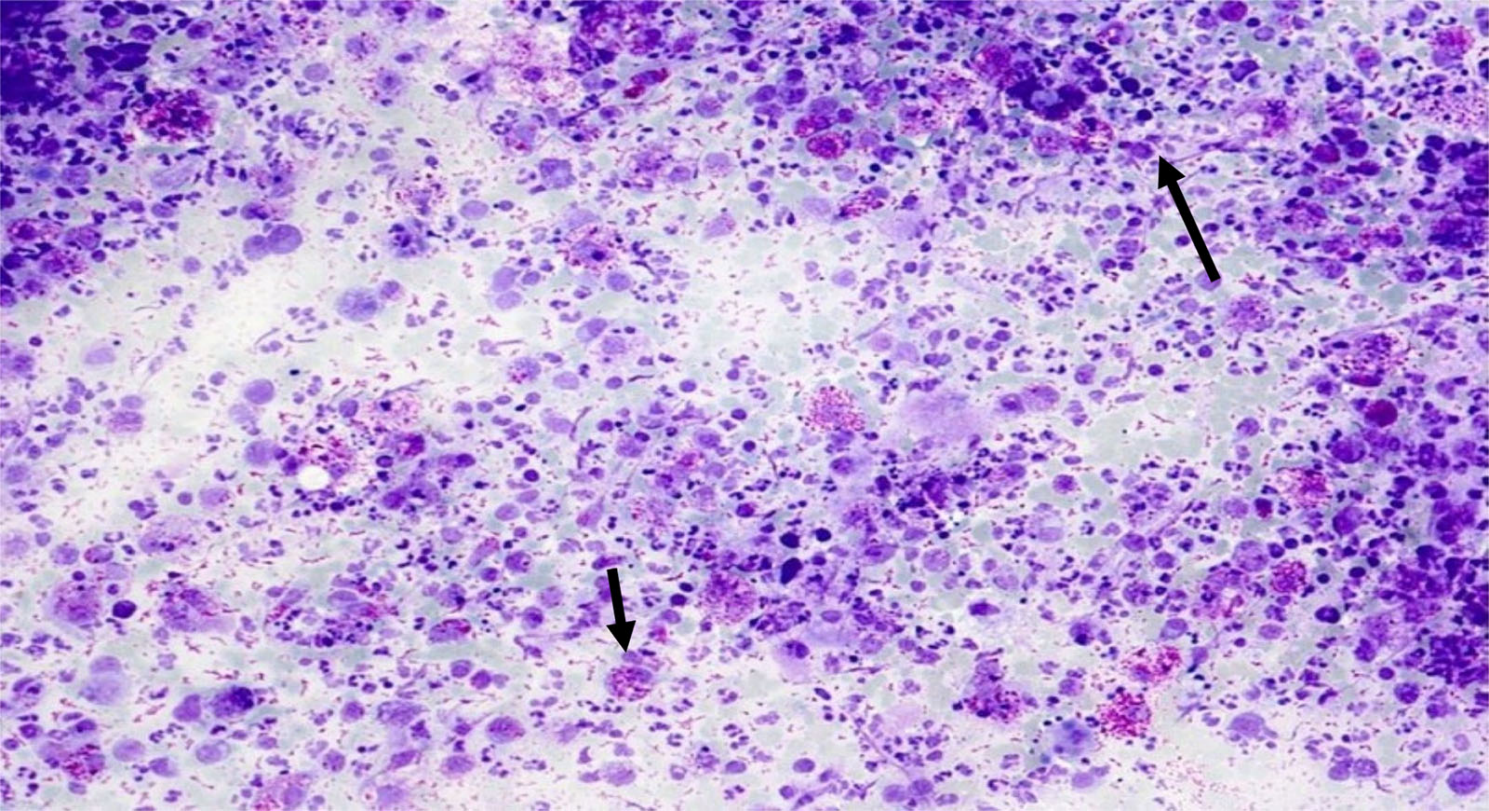
Acute pancreatitis with necrotic cell clusters, cell debris and dirty background (long arrow). Acinar and ductal cells, occasionally degenerative altered without nucleoli (small arrow).

A: **Acute pancreatitis**: The cytological slides showed necrotic cell clusters with cell debris, dirty background and inflammatory cells, including macrophages and neutrophils (Figure 3). There may be calcification and fatty tissue necrosis. Acinar and ductal cells, occasionally degenerative altered cells will be seen but without nucleoli. The cells showed pyknotic nuclei with karyorrhexis. The nuclei are with dense, pyknotic and condensed chromatin.

The etiology can be metabolic, vascular, mechanical, or infectious. Their pathogenesis arises through different mechanisms. The frequency is female: male = 1:1, female = 50–70 years, male: 30–50 years, with incidence in west countries of 13–100/100 000 [12, 13]. The complications of acute pancreatitis are for example formation of pseudocysts with calcifications.

**Figure 4:**
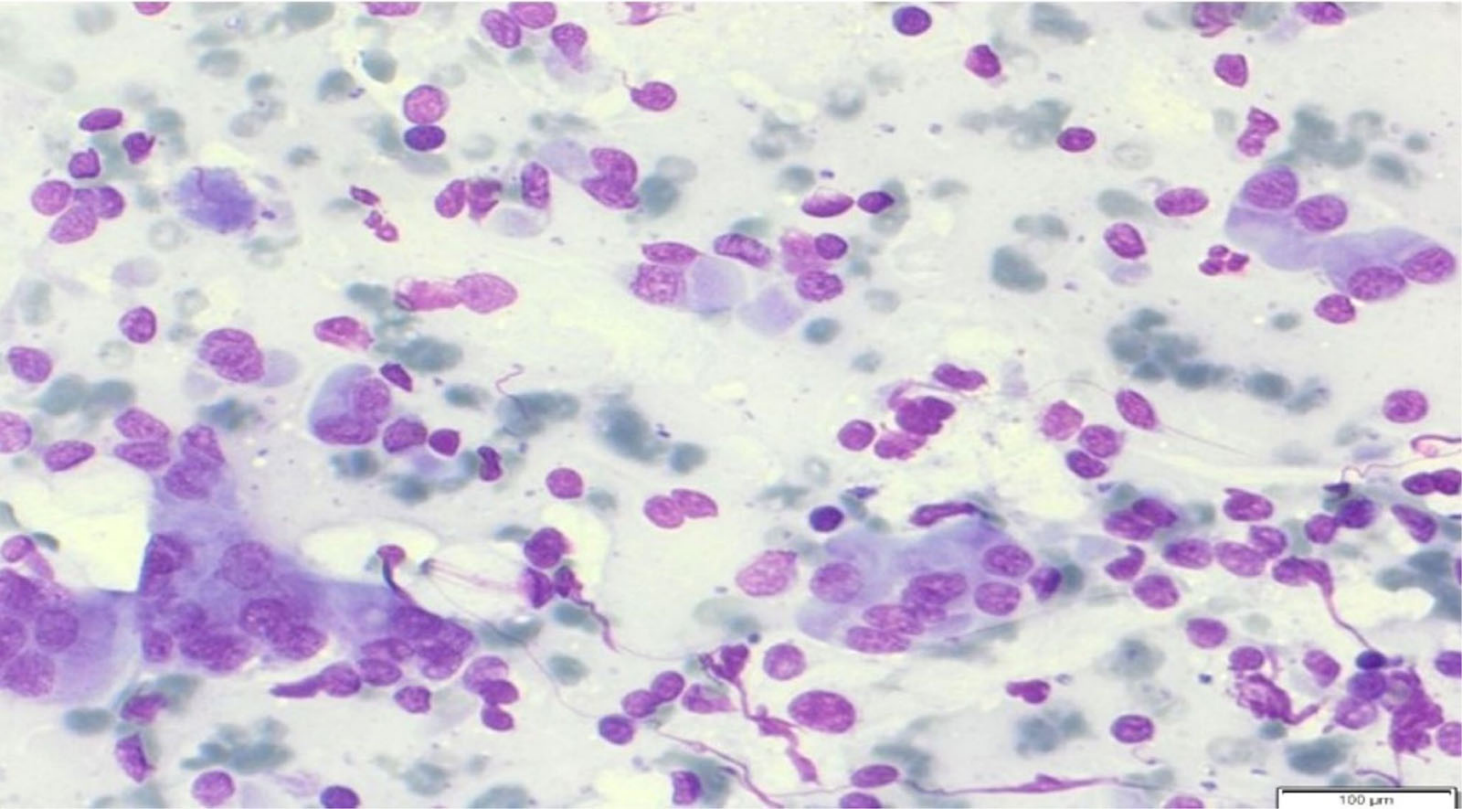
Chronic pancreatitis with variable groups of cells in shape and size. Acinus and ductal epithelium in regular cell groups or in “clusters” with regenerative changes. Enlarged nuclei and maybe with prominent nucleoli.

**B: Chronic pancreatitis:** The cytological slides showed acinus and ductal epithelium in regular cell groups or in “clusters” with regenerative changes (Figure 4). The cells are with variable, bright and vacuolated, sharply demarcated cytoplasm. The nuclei are with granular and hyperchromatic chromatin and enlarged nucleoli. The background is very variable. Many inflammatory cells are seen like lymphocytes, plasma cells, fewer granulocytes. Other cells like fibroblasts and connective tissue can also be found.

It develops slowly through recurrent acute pancreatitis and cystic fibrosis or parasitosis. Many factors play a role in its formation, for example, biliary hypercalcemia (gallstones) or alcohol and tobacco smoking. The frequency is female: male = 1:9, at the 30th–60th year of life. The complications of chronic pancreatitis are pseudocysts, duct obstruction due to scar strictures, lithiasis, malabsorption, diabetes mellitus, ascites and pleural effusions.

Differential diagnosis: Cytological differentiation from pancreatic carcinoma can be very difficult, here, cell clusters with cell atypia in three‐dimensional clusters are often identified with overlapping nuclei and a coarser chromatin structure.

4

**Cystic Pancreatic Lesions:** These are different subtypes.

Pseudocysts (posttraumatic/postinflammatory) are more common than true cysts (congenital = 10%–15% or acquired = 70%–90%, slow growth). The incidence reaches about 24.3% in autopsy material and increases with age and diffusely distributed in the pancreas [14].

Types:

1. Serous cystadenoma (benign).
2. Mucinous cystic neoplasia (mucin‐positive, high potential for degeneration, low and high‐grade) [15].
3. Intraductal papillary mucinous neoplasia = IPMN (affects the entire organ and has a better prognosis than ductal carcinoma [16, 17, 18].
4. Pseudopapillary neoplasia, which affects women from their 4th decade of life, malignant degeneration maybe occurred.

Rare forms:

1. Cystic teratoma
2. Cystic choriocarcinoma
3. Parasitic cysts
4. Polycystic pancreas
5. Enterogenous cyst
6. Lymphoepithelial cyst
7. Cystic carcinoma
8. Endometrial cyst
9. Branchiogenic cyst
10. Dermoid cyst

CAVE: Differential diagnosis: Cystic degenerated ductal pancreatic carcinoma.

Mucinous are not benign but serous are benign.

Cytology of a serous cyst (10% of all cysts): The cytological slide showed epithelial cell aggregates (lining of the cells), cell detritus with inflammatory cells.

Differential diagnosis: neoplastic pancreatic cyst, here one finds more cell debris, naked nuclei with atypia and occasional necrosis.

Cytology of the pseudocyst (90% of all cysts): There is cloudy or clear liquid with inflammatory reaction. In the background, there are erythrocytes/fibroblasts, cell detritus, and rarely activated mesothelium.

**Figure 5:**
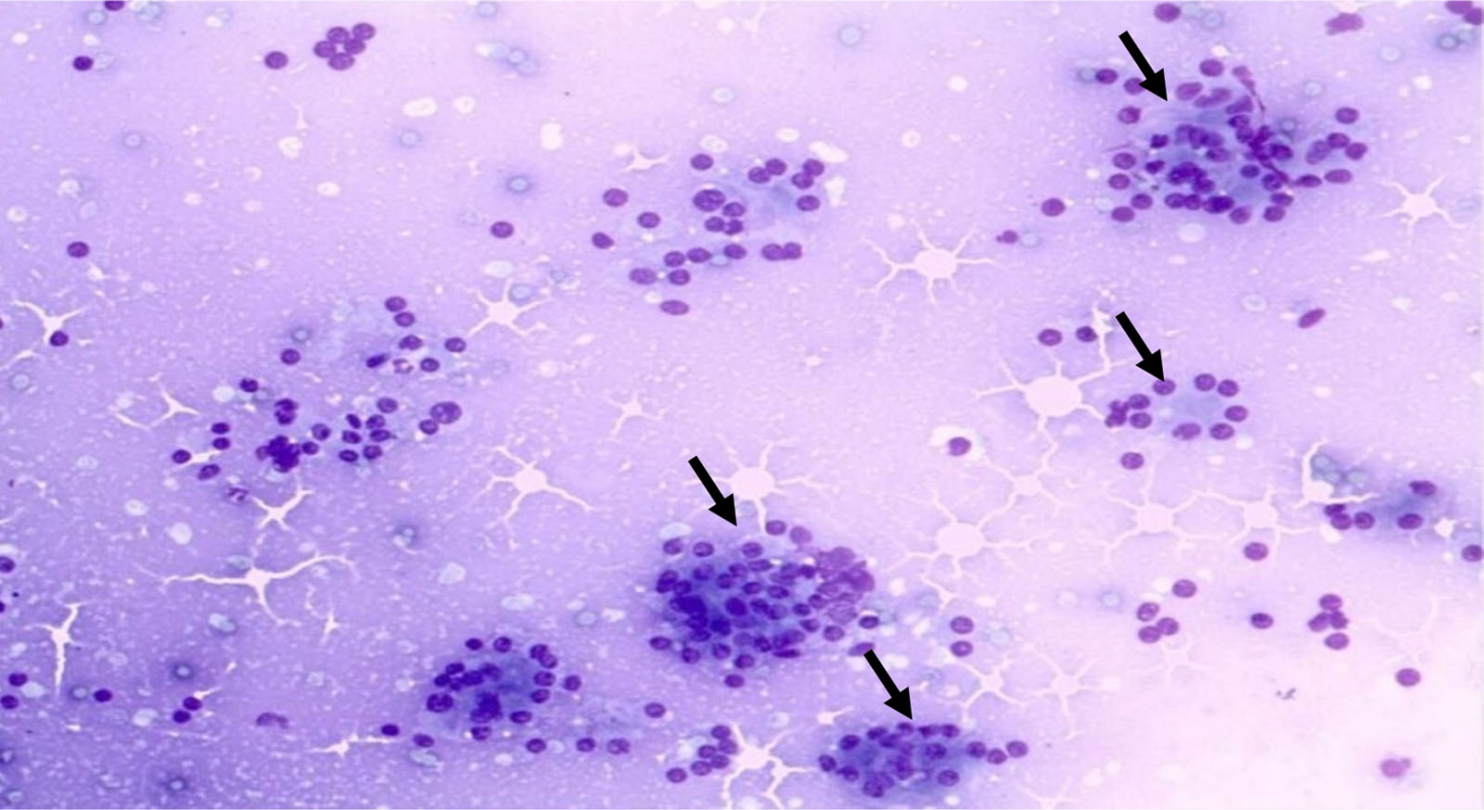
Serous cystadenoma with little number of bland‐shaped cells with cleanly background. Cylindrical epithelium in honeycombed structures. Round, uniform and central nuclei (arrows). No nucleoli. Broad, light blue‐stained cytoplasm.

Cytology of serous cystadenoma: There is a little number of bland‐shaped cells with clean background. There are cylindrical epithelial cells in honeycombed structures. The cells showed round, uniform and central nuclei, with regularly distributed chromatin and without nucleoli. They have broad, light‐blue‐stained cytoplasm (Figure 5).

**Figure 6:**
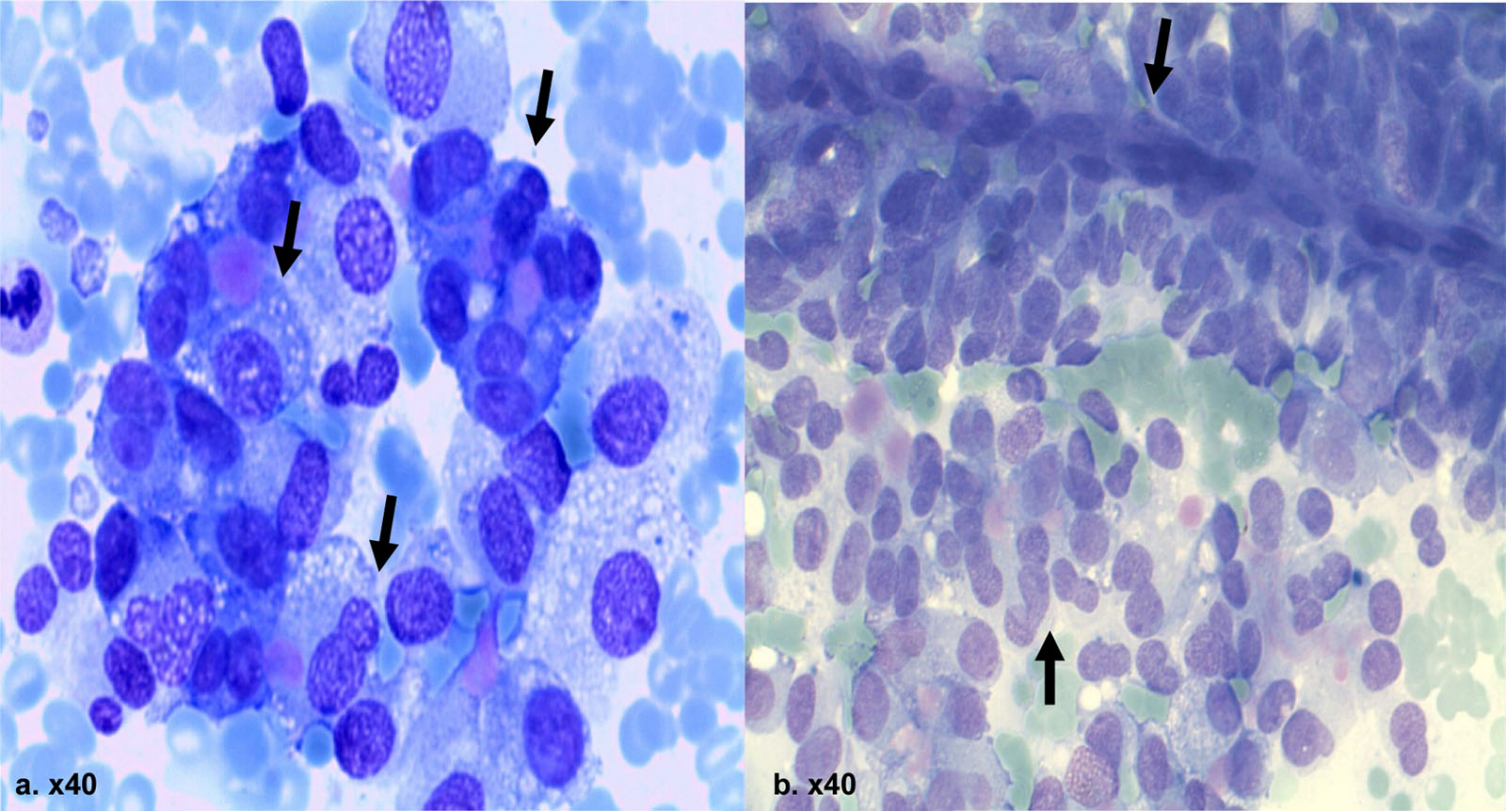
Intraductal papillary mucinous neoplasia with moderate dysplasia.

Borderline tumors will include mucinous cystic neoplasia with moderate dysplasia, intraductal papillary mucinous neoplasia with moderate dysplasia (Figure 6) and solid pseudopapillary neoplasia.

## 5 Cytological Criteria of Pancreatic Samples for Different Malignant Pancreatic Lesions Based on Our Experience

### 5.1 Cytology of Pancreatic Carcinoma

**Figure 7:**
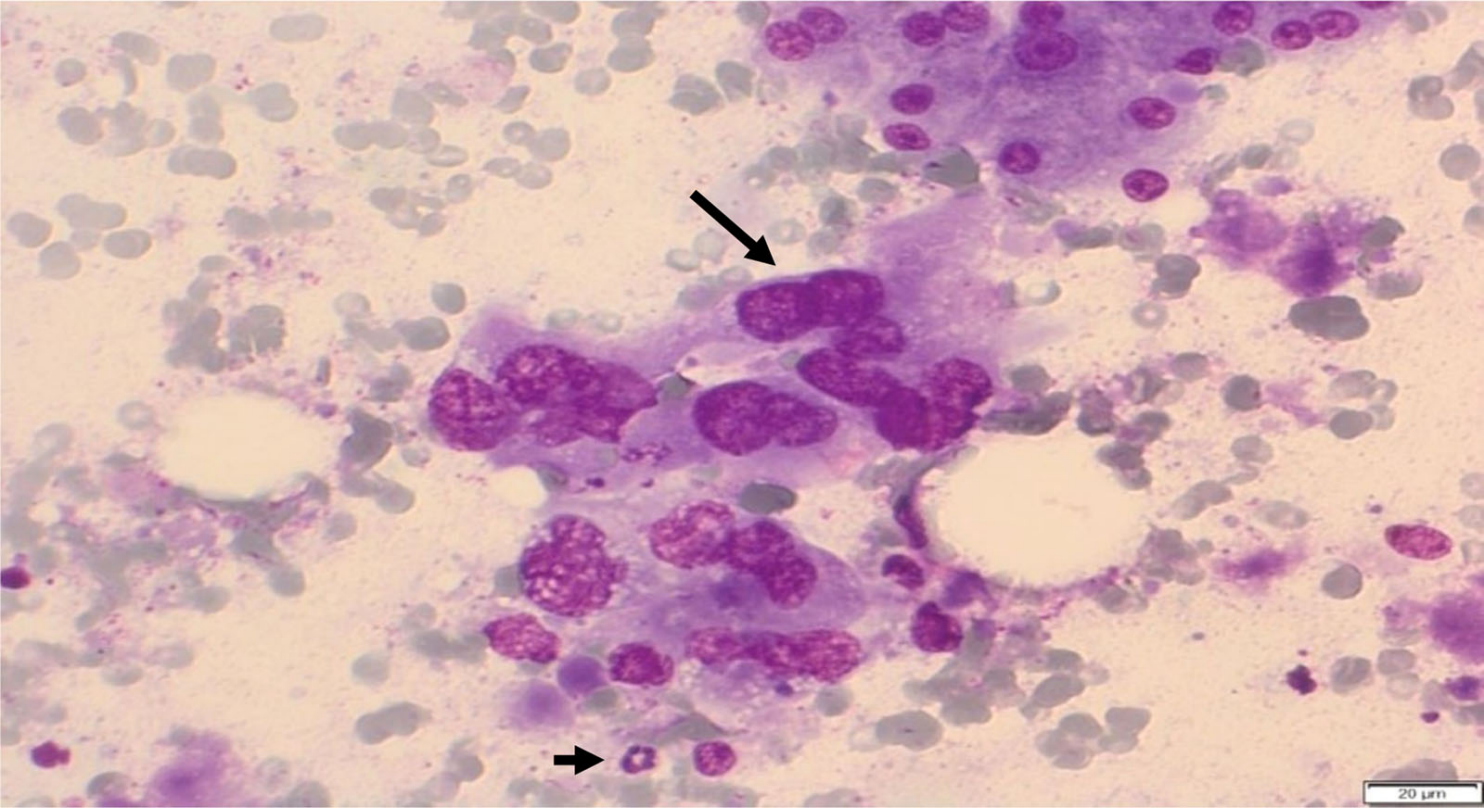
Ductal adenocarcinoma with neoplastic cells (long arrow), which lie isolated in glandular formations or papillary groups with coarse and irregularly distributed chromatin. Inflammation, necrosis, cell phagocytosis, dirty background (small arrow). Cell and nuclear pleomorphism, strong anisokaryosis with prominent nucleoli and mitoses.

The cytological slides showed cell and nuclear pleomorphism with strong anisokaryosis. These are isolated or lie in glandular formations or solid groups. The cells showed enlarge nucleus with frequent mitoses and nuclei. The nuclei showed coarse and irregularly distributed chromatin. There are inflammatory cells, necrosis and dirty background. Stroma cells may be seen as a sign of desmoplasia (Figure 7).

Pancreas carcinoma is the fourth most common cause of cancer‐related death in Germany with around 14,000 deaths per year. In USA, there is incidence between men of 57/100,000 and between females of 41/100,000 [19]. In Germany, there is incidence within men of 12,270/100,000 and within females 9960/100,000 [20]. It is most commonly located in the head of the pancreas and is characterized by aggressive growth behavior with early metastasis. At the time of diagnosis only 20% tumors are resectable tumors allowing a curative R0 resection (e.g., Whipple operation) with subsequent adjuvant chemotherapy. The age peak is in the 7th and 8th decade. Since the 1930s, the incidence has nearly doubled in the United States and most western countries. 2/3 of all patients are older than 60 years and rarely under 45 years. The carcinomas are ductal adenocarcinomas (90% of all pancreatic neoplasms and with a poor prognosis), followed by neuroendocrine carcinomas and non‐ductal carcinomas (cystadenocarcinomas and acinar cell carcinomas) [21, 22]. Cigarette smoking, alcohol consumption and occupational chemical toxins as well as dietary factors and last but not least genetic factors play an important role in the pathogenesis. The incidence of pancreatic ductal adenocarcinoma is increasing slightly. The prognosis for patients with pancreatic cancer remains poor. Early detection is difficult [5, 23, 24, 25].

CAVE: Differential diagnosis: chronic pancreatitis.

### 5.2 Grading of Ductal Carcinomas

The grading of this carcinoma is divided into three grades and correlates with the glandular differentiation, amount of mitosis, mucin staining and cell pleomorphism [26]:

Severe differential diagnosis: other pancreatic tumors and adenocarcinomas of other locations, especially cholangiocellular carcinoma (CCC). Immunocytochemistry is not always helpful; CK7/CK20 are not always positive (only 50% of the time). The morphology in pancreatic cytology is the basis for diagnosis.

Rare tumors of the pancreas:

1. Mucinous noncystic carcinomas (1%–3%).
2. Signet ring carcinoma (1%).
3. Adenosquamous carcinoma (4%–3%).
4. Undifferentiated/anaplastic carcinomas (2%–7%) and
5. Osteoclast‐like giant cell tumors ( < 1%).

### 5.3 Acinar Cell Carcinoma

**Figure 8:**
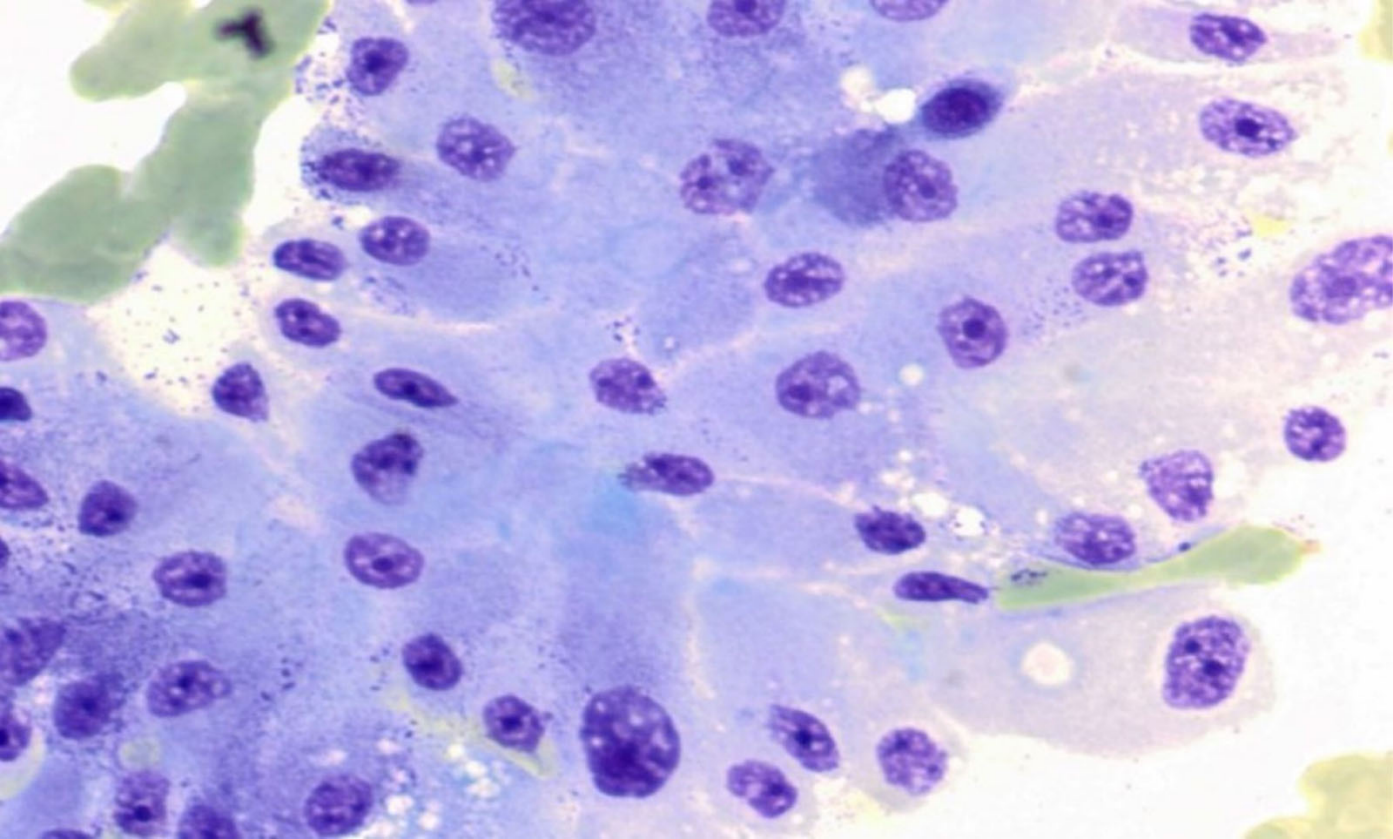
Acinar cell tumor: rounded, angular, cylindrical Cells, arranged in acinar structures, basally located nuclei and Eosinophilic granulated cytoplasm.

The cytological smears showed rounded, angular or cylindrical cells. They are arranged in acinar structures with basally located nuclei and eosinophilic granulated cytoplasm (Figure 8). The incidence of these tumors is 1%–2% of exocrine carcinomas, rarely multicentric [26]. The average age is approximately 60 years old. Males are more commonly affected [27]. Immunocytochemistry is a key diagnostic role by reactivity to antibodies of trypsin, chymotrypsin, lipase and amylase [27, 28, 29]. The survival of patients with acinar cell carcinoma is poor, with a median survival time of 19 months and a 5‐year survival rate of 25% [27].

### 5.4 Endocrine Tumors

The cytological slides showed cell rich preparation. The cells are cubic, slightly spindle‐shaped. The nuclei are monomorphic with granulated chromatin and commonly visible nucleoli. The cytoplasm is eosinophilic or cyanophilic.

Classification of pancreatic neuroendocrine tumors (NET) entity (according to WHO 2019) [30] and Ki‐67‐based grading (Table 1).

**Table 1:** WHO‐classification of NET and NEC according to mitoses and Ki‐67.

Entity with Grading	Ki‐67	Mitoses/2 mm^2^
NET G1	< 3%	< 2
NET G2	3%–20%	2–20
NET G3	> 20%	> 20
NEC	> 20%	> 20

Mixed tumors (“mixed neuroendocrine non‐neuroendocrine neoplasm”).

CAVE: The decisive factor is the immunocytochemistry/immunohistochemistry positivity for: NSE, Chromogranin, Synaptophysin, INSM1‐A‐8.

**Figure 9:**
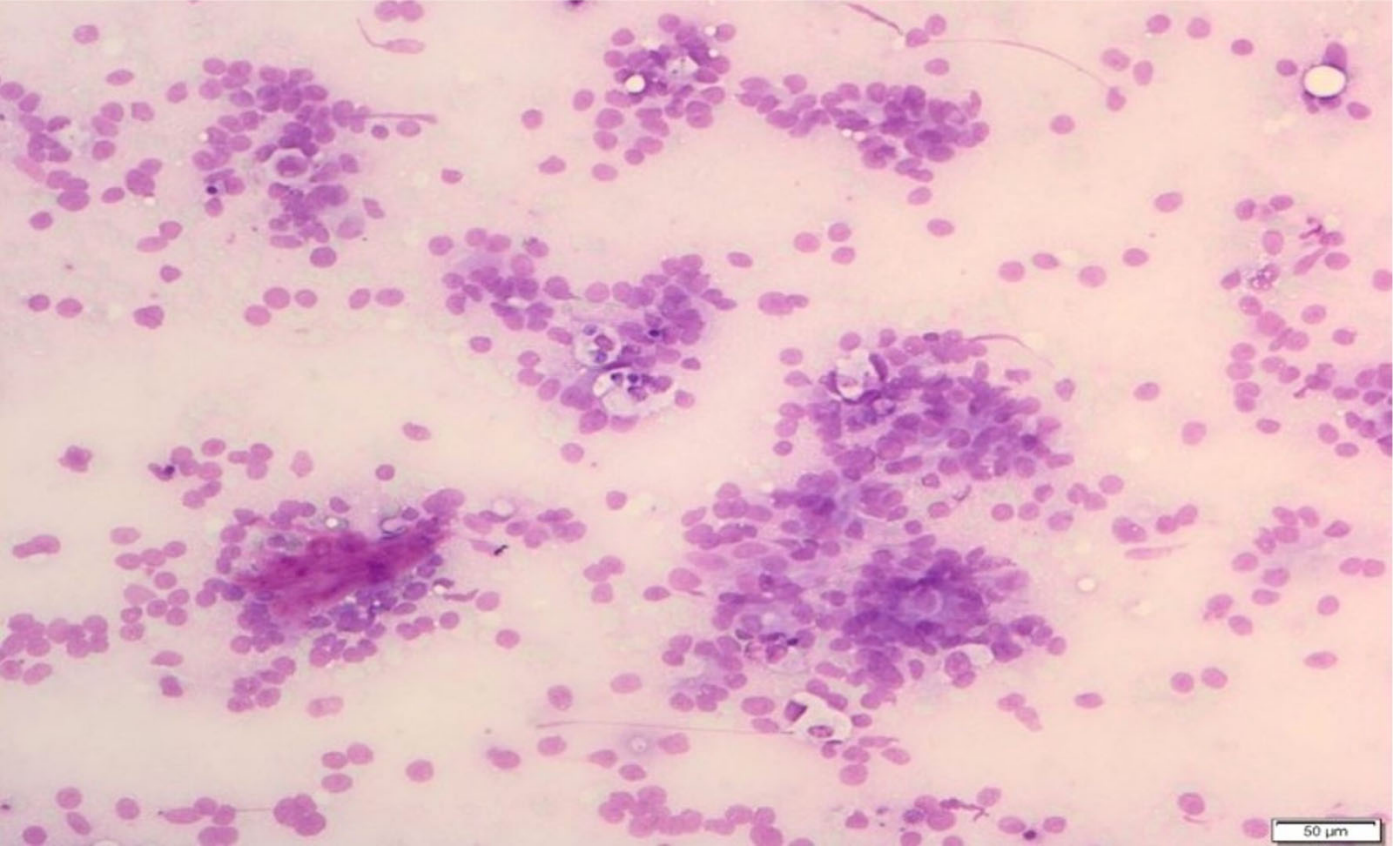
Small cell neuroendocrine carcinoma: cell‐rich preparation with cubic, slightly spindle‐shaped cells and monomorphic nuclei as well as granulated (salt and pepper pattern) chromatin and visible nucleoli. Cyanophilic or eosinophilic cytoplasm.

Differential diagnosis: neuroendocrine tumors in other locations (Figure 9) (Table 2).

**Table 2:** Cell type of pancreas tumors.

Cells of origin	localization	tumor entity	tumor marker
Ductal	cells large ducts	Mucinous tumors	CEA +
Ductular cells	Small ducts	Serous tumors	CEA ‐
Acinar cells	Acini	Acinar cell tumor	Trypsin +, Lipase +, α1‐Antitrypsin +
Endocrine cells	islets of Langerhans	islet cell tumor	Chromogranin +, NSE +, Synaptophysin

### 5.5 Metastases in the Pancreas

**Figure 10:**
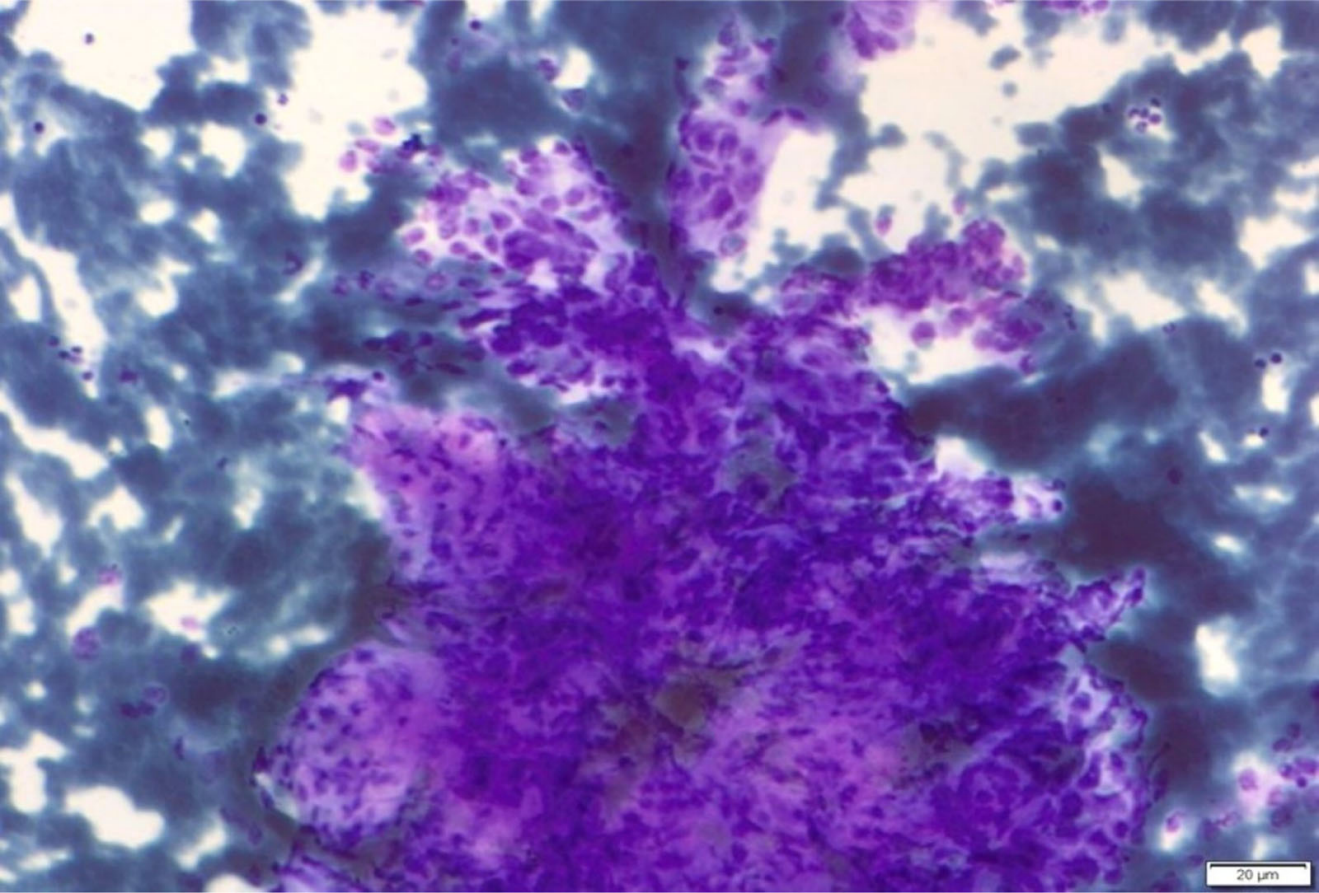
Metastasis from renal cell carcinoma in the pancreas with clear cell groups and interstitially endothelial cells of the capillaries.

Metastases in the pancreas are not common; bronchial and renal cell carcinomas are the most common (Figure 10) (Table 3).

**Table 3:** Primary origin of metastases (*N* = 21 [8.1%]).

Primarius	Number
Renal cell carcinoma	5
Bronchial cell carcinoma (large cell)	4
Bronchial cell carcinoma (small cell)	2
Non‐Hodgkin‐lymphoma	3
Breast cancer	3
Adrenal carcinoma	1
Medullary carcinoma of the thyroid gland	1
Non‐seminomatous germ cell tumor	1
Seminoma of the testicle	1

## 6 Molecularpathological Background of Pancreas Ductal Adenocarcinoma

More than 90% of pancreatic malignancies are ductal pancreatic carcinomas, which gradually develop from premalignant precursors, so‐called PanIN (pancreatic intraepithelial neoplasia). There are four main driver genes *KRAS, CDKN2A, TP53* and *SMAD4*. Oncogenic point mutations of the *KRAS* gene can be detected in > 90% of pancreatic carcinomas and, along with the alterations for *CDKN2A*, are among the early events in carcinoma development. The *KRAS* status has a prognostic meaning; it has been shown that pancreatic carcinoma with wild‐type status for *KRAS* show a better course of the disease. Up to 10% of pancreatic carcinomas are hereditary/genetic, with the following syndromes and genes playing a role: hereditary breast and ovarian carcinoma (*BRCA1* or *BRCA2* gene), hereditary non‐polypoid colon carcinoma (*HNPCC*, mismatch‐repair gene), ataxia telangiectasia (*ATM* gene), familial adenomatous polyposis (*FAP, APC* gene), Peutz‐Jeghers syndrome (*STK11* gene), familial atypical multiple mole–melanoma (FAMMM)‐pancreatic carcinoma syndrome (FAMMMPC syndrome, *CDKN2A* gene). The detection of germline mutations for the genes listed is not only important for the inclusion of affected family members in screening programs, but also represents a predictive marker for the clinical efficacy of PARP inhibitors (PARP = Poly (ADP‐ribose) polymerase)), which is why a human genetic consultation should be recommended to patients with pancreatic carcinoma. The molecular (*KRAS* and/or *GNAS* mutation) findings on the cyst fluid are part of the formal diagnostic criteria of neoplastic cysts [31, 32, 33, 34, 35, 36, 37, 38, 39, 40, 41, 42, 43, 44, 45, 46].

## 7 Materials and Methods

Endosonography is a method of endoscopic ultrasound that has been established in the mid‐1990s, with the transducer being inserted into the body. The transducer is located very close to the target structure, making it possible to detect the smallest pathological changes. Ultimately, it is a technical combination of gastroscopy and ultrasound. This examination is particularly important for assessing the pancreas. Percutaneous ultrasound‐guided for larger, CT‐guided for smaller masses. FNA with 0.7–0.9 mm gauge [8]. Only cytological smears have been finished and stained with MGG (May‐Grünwald‐Giemsa). Immunocytochemistry was performed in metastases and neuroendocrine tumors as well as unclear cases. In some cases, the performance of immunocytochemistry was evaluated with the help of histology. A total of 1290 samples from fine needle aspiration cytology of pancreas lesions were processed in the Institute of Pathology at Hannover Medical School (MHH) between 1998 and 2023, and stained with Giemsa and PAS stains as conventional methods. Exocrine pancreatic tumors with cytological classification (MHH) were identified in 259 patients (*N* = 259), (Table 4). These were compared with the histological specimens that were processed at the same institute (Table 5). The molecular pathology was done in some samples in the institute with next generation sequencing (Ion Torrent‐Technology). The local ethics committee has stated that, there is no need for approval due to the retrospective nature of the study.

**Table 4:** Exocrine pancreatic tumors, cytological classification, (MHH) (N = 259).

Tumor entity	*N*	%
Ductal carcinoma	176	67.95
Mucinous, noncystic carcinoma	6	2.31
Adenosquamous carcinoma	11	4.24
Undifferentiated carcinoma	13	5.01
Intraductal papillary carcinoma	4	1.54
Neuroendocrine tumors (NETs)	27	10.42
Acinar cell carcinoma	1	0.38
Metastases*	21	8.10
Total	259	100

* Metastases were diagnosed purely morphologically and confirmed by immunocytochemistry.

**Table 5:** Cases of the pancreas lesions (1998–2023) with the cytological diagnosis, Male: Female = (762 [59%]:528 [41%]).

Diagnosis	Number	%
Inconspicuous/Inflammation	817	63,333
Pseudocysts/cysts	96	7.441
Malignant	259	20.077
Nonrepresentative	118	9.147
Total	1290	100

## 8 Results

Insufficient material was collected in 10% (9.147%) of the samples. Complications were observed rarely, in about 1% of cases. For accuracy, we compared the results between the FNP and the final histopathological diagnoses from the resection and calculated the specificity and sensitivity of this method to detect neoplastic and non‐neoplastic lesions. The agreement between FNP and histology of the carcinomas was 97.09% with a false positive rate of 0.97% (ductal carcinoma) and a false negative rate of 1.94% (two carcinomas misdiagnosed). The sensitivity was 98% and the specificity 99% (Table 4). Different grades have been found within our cases (73). Well‐differentiated (G1) ductal carcinoma has been found in 5/73 (6.84%) cases. Moderately differentiated (G2) ductal carcinoma has been found in 21/73 (28.76%) cases. Poorly differentiated (G3) ductal carcinoma has been found in 27/73 (37%). Transitional form of G2–G3 = 20/73 (27.4%).

## 9 Summary and Conclusion

Pancreatic FNA is the gold standard, its power is strongly associated with high specificity and sensitivity in the diagnosis of pancreatic lesions, and is very useful in the differential diagnosis between malignant and inflammatory lesions in pancreas. The smears are very suitable for molecular genetic studies. Cytology can reduce the number of unnecessary operations.

## Author Contributions

B.S. developed the idea of the work and study design, interpreted the results and drafted the manuscript. M.A. interpreted the results and provided final approval. All Authors have read and approved the final version of the manuscript.

## Ethics Statement

It is stated that, there is no need for the approval, due to the retrospective nature of the study (Ethics Committee of the medical association, Hannover, Germany).

## Consent

The authors have nothing to report.

## Conflicts of Interest

The authors declare no conflicts of interest.

## Transparency Statement

The lead author Abbas Mahmoud affirms that this manuscript is an honest, accurate, and transparent account of the study being reported; that no important aspects of the study have been omitted; and that any discrepancies from the study as planned (and, if relevant, registered) have been explained.

## Data Availability

The data set used and/or analyzed during the current study are available from the first and corresponding authors upon reasonable request. They take the complete responsibility for the integrity of the data and accuracy of the data analysis.
